# Outcomes and Complications of Percutaneous Nephrolithotomy (PCNL): A Single-Center Experience

**DOI:** 10.7759/cureus.69567

**Published:** 2024-09-17

**Authors:** Mazhar Ali, Qazi Naeem, Umair Zafar, Ansar Abbas, Faizan Muhammad, Muhammad Naqash, Nida Ghouri, Murad A Khan, Abdullah Ali

**Affiliations:** 1 Urology, Recep Tayyip Erdoğan Hospital, Muzaffargarh, Muzaffargarh, PAK; 2 Research, Indus Hospital and Health Network, Karachi, PAK; 3 General Surgery, Shifa International Hospital Islamabad, Islamabad, PAK; 4 Surgery, Shifa College of Medicine, Shifa Tameer-e-Millat University, Islamabad, PAK

**Keywords:** bull's eye technique, percutaneous nephrolithotomy (pcnl), stone clearance, stone-free rate, surgical techniques, urolithiasis

## Abstract

Background: Urolithiasis is extremely prevalent in Pakistan, with percutaneous nephrolithotomy (PCNL) emerging as the primary treatment modality over traditional open surgery. Despite its effectiveness, PCNL is associated with a risk of complications and residual stones. There is limited data on PCNL outcomes from Southern Punjab, necessitating an evaluation of its efficacy and safety in this region.

Methods: We conducted a retrospective analysis of 399 patients who underwent PCNL at a tertiary care hospital in Muzaffargarh, Pakistan, between October 2016 and September 2022. Detailed preoperative assessments, surgical procedures, and postoperative outcomes were reviewed. Stone clearance and complication rates were assessed, and factors influencing these outcomes were analyzed.

Results: The median age of the study population was 39 years, with a male predominance. Stone clearance was achieved in 80.45% (321) of cases, with higher success rates observed in lower pole punctures. Complications occurred in 2% (13) of patients, predominantly hydrothorax following upper pole puncture. Patients with comorbidities had a higher risk of complications (P = 0.097). Residual stones were more common in staghorn stones and larger stone sizes (>3-4 cm). The median operative time was 60 minutes, shorter than reported in the literature, reflecting surgical expertise.

Conclusion: PCNL is an effective and safe treatment option for urolithiasis in Southern Punjab, Pakistan, with favorable stone clearance rates and low complication rates. Tailoring treatment strategies based on patient characteristics and optimizing surgical techniques are essential for improving outcomes in this population.

## Introduction

Urolithiasis ranks as the sixth most prevalent condition in Pakistan, a country situated within the "stone belt" region [1]. Historically, open surgery (pyelolithotomy) has served as the primary approach for treating renal stones, but endoscopic procedures, particularly percutaneous nephrolithotomy (PCNL), are increasingly gaining favor as the preferred therapeutic modality [2-5]. PCNL demonstrates superior efficacy in achieving stone-free outcomes compared to retrograde intrarenal surgery (RIRS) and extracorporeal shock wave lithotripsy (ESWL), particularly for larger and staghorn stones [6]. Literature reveals a complication rate of approximately 10% associated with these interventions, encompassing complications such as hemorrhage, hydrothorax (following upper pole puncture), injury to the collecting system, urosepsis, fluid overload, nephrocutaneous fistula, and rarely, colonic injury [7,8]. Stone clearance rates post-PCNL range between 95.3% and 97.3% [9]. As per guidelines outlined by the European Association of Urology, PCNL stands as the recommended first-line treatment for large (>2 cm), multiple, and inferior calyx renal stones [10-12].

Despite the prevalence of PCNL in the management of urolithiasis, there remains a paucity of published literature detailing outcomes specifically from the South Punjab region of Pakistan. Thus, the present study endeavors to assess and elucidate the outcomes of PCNL procedures conducted by a single surgical unit at Indus Hospital, Muzaffargarh, Pakistan. Notably, puncture techniques for the upper and lower poles have been implemented utilizing distinct methodologies, including the bull’s eye technique for the upper pole and either the bull’s eye or uniplanar technique for the lower pole. The bull’s eye technique in PCNL is a fluoroscopy-guided method for accessing the kidney. It involves aligning the needle with the target calyx under fluoroscopic imaging, creating a "bull’s eye" view. The needle is advanced towards the stone in a straight line, minimizing deviation. This technique enhances precision and reduces complications. It is especially useful for obtaining an optimal percutaneous tract to the kidney stone.

## Materials and methods

We conducted a retrospective analysis of the medical records of 399 patients who underwent PCNL between October 2016 and September 2022 at the Recep Tayyip Erdoğan Hospital, Muzaffargarh, a tertiary care facility. We included the patients who underwent PCNL between October 2016 and September 2022 at the Recep Tayyip Erdoğan Hospital, Muzaffargarh. Patients who were assessed and deemed fit for surgery following anesthesia risk assessments i.e., American Society of Anesthesiologists (ASA) 1 and 2 were made part of this study. Patients who provided a sterile urine culture report before surgery and patients who underwent comprehensive preoperative assessments, including laboratory and radiological evaluations, were included in the study. We excluded the patients who were not deemed fit for surgery following anesthesia risk assessments and excluded ASA 3 and 4. The patients who had urinary tract infections (UTIs) and those who underwent procedures other than PCNL were also excluded from this analysis. The patients with incomplete medical records and those who did not adhere to follow-up were also removed from this study. Postoperative patients were graded using the Clavien-Dindo classification system and those classified as Grade I or II were omitted from the study.

A comprehensive assessment was undertaken, encompassing detailed medical histories, physical examinations, and laboratory investigations comprising complete blood counts (pre- and post-operative), serum creatinine levels, coagulation profiles, serum electrolyte levels, urinalyses, and urine cultures with sensitivity testing. Radiological evaluations involved ultrasound of the kidneys, ureters, and bladder (KUB), supplemented by intravenous pyelography or non-contrast computed tomography (CT KUB) where necessary. Follow-up examinations aimed to detect residual stones, utilizing X-ray KUB for radio-opaque stones and predominantly ultrasound for radiolucent stones, with X-rays reserved for certain radio-opaque cases. Residual stone fragments are generally defined as stone fragments remaining in the urinary system after the completion of an intervention (ESWL, ureteroscopy (URS), or PCNL).

Prior to surgery, patients underwent anesthesia risk assessments and were deemed fit for the procedure. Surgical bookings were contingent upon the receipt of a sterile urine culture report (indicating negative urine culture and sensitivity) for each patient. Those presenting with UTIs received treatment from the infectious disease department 7-10 days before proceeding with surgery. The study protocol received approval from the institutional review board and adhered strictly to ethical standards. It was approved in the year 2022 and the study IRB number is IHHN_IRB_2022_12_024.

PCNL technique

All procedures were conducted under general anesthesia. A 5-French ureteric catheter was cystoscopically inserted into the renal collecting system under fluoroscopic guidance. If a stone was lodged at the pelvic level, a ureteropyelography was performed to maneuver the guide wire beyond the stone, facilitating the subsequent passage of the ureteric catheter under fluoroscopic guidance. Subsequently, the patient was positioned prone, and retrograde pyelography was performed to delineate the pelvicalyceal system and determine the puncture site. The target calyx was punctured with an 18-gauge needle under fluoroscopic guidance, utilizing either the bull’s eye technique for the upper or lower calyx or the triangulation/parallel technique for the lower calyx. Once the puncture was confirmed, a hydrophobic guidewire was directed into the ureter or the desired calyx. In cases where multiple tracts were anticipated, multiple punctures were made, and wires were passed through each tract. The tract was initially dilated with fascial dilators up to 10 Fr, followed by serial dilation using Alkens metallic dilators (France) over the olive tip, under fluoroscopic guidance, up to 24 or 30 Fr. An appropriately sized Amplatz sheath (Cook Medical, Bloomington, USA) was then inserted. A rigid nephroscope was introduced to visualize and fragment the stones using pneumatic lithotripsy (EMS). Stone fragments were retrieved using grasper forceps, or in the case of multiple small fragments, by flushing with a 12 Fr Nelaton catheter (Cook Medical, Bloomington, USA) inside the Amplatz sheath. A 4.7-Fr double-J stent was placed if stone clearance was incomplete or in cases of staghorn stones. Nephrostomy was maintained when deemed necessary (using an 18-22 Fr Foley catheter). On the first postoperative day, chest auscultation was performed for patients who underwent upper pole puncture, with a chest X-ray recommended if decreased breath sounds were noted, to rule out hydrothorax. Nephrostomy tubes were typically removed on the first or second postoperative day, regardless of stone-free rates (SFRs), as second-look PCNL was not routinely performed. Complications were graded according to the Clavien classification system, we only included the patients who were classified under Grade IIIa, IIIb, IVa, IVb, and V, with modifications specific to percutaneous procedures.

## Results

Throughout the study duration, a total of 399 patients underwent the PCNL procedure. The median age of the study population was 39 years (interquartile range, IQR: 29-50), demonstrating a male predominance. Detailed demographic characteristics and comorbidities of the patients who underwent PCNL are presented in Table 1.

**Table 1:** Basic demographics and co-morbid of patients undergoing PCNL (N=399) PCNL: percutaneous nephrolithotomy

Category	N (%)
*Gender*	
Male	220 (55.13)
Female	179 (44.86)
*Stone laterality*	
Right-sided stone	203 (50.87)
Left-sided stone	196 (49.12)
*Co-morbidities*	
Hypertension	29 (07.26)
Diabetes mellitus	16 (04.01)
Others	45 (11.27)
None	297 (74.43)

**Figure 1:**
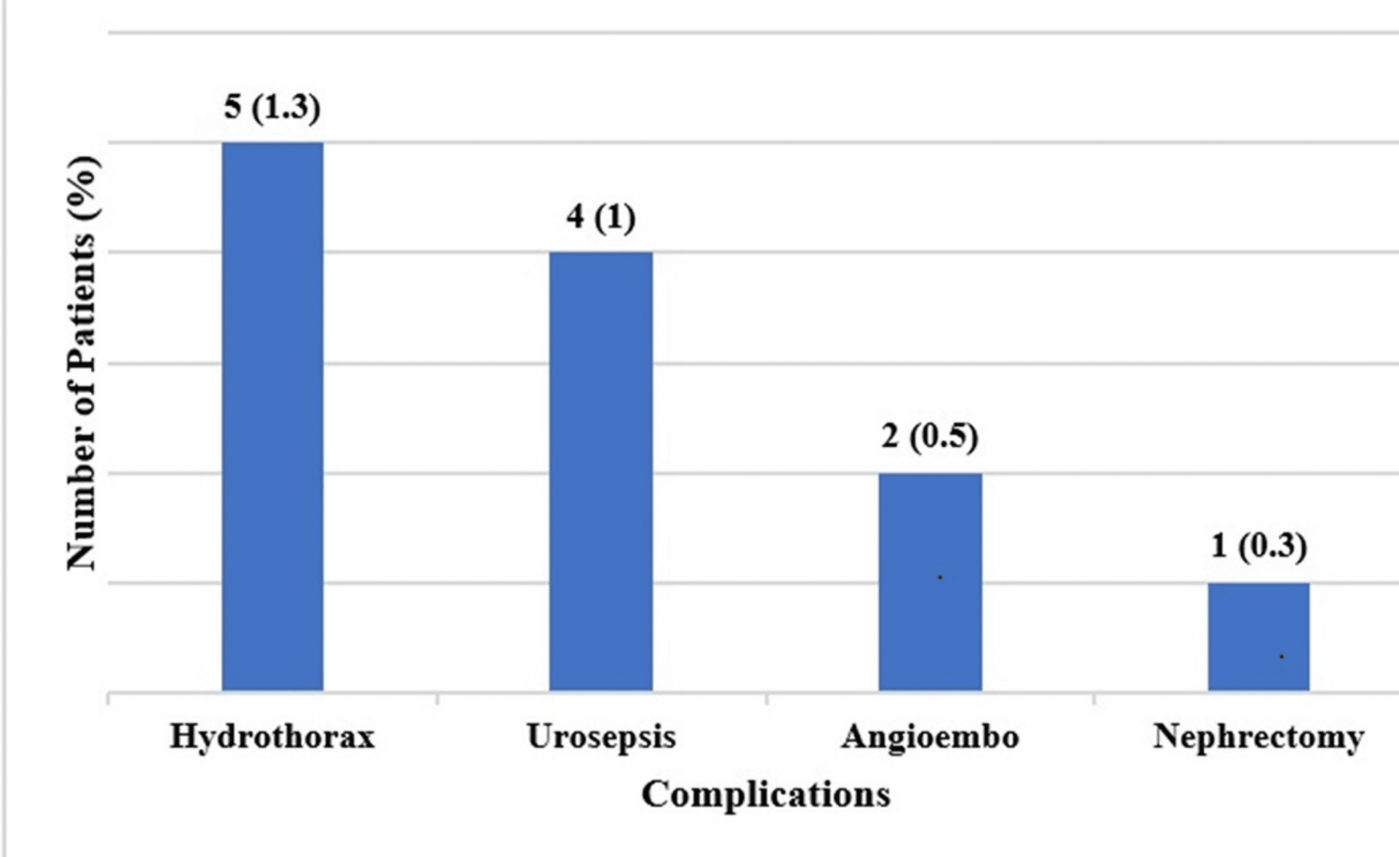
Bar chart summarizing the complications developed in patients

The primary outcomes of the study encompassed the occurrence of complications and the presence of residual stones, as delineated in Tables 2-3, respectively. Remarkably, only 2% of the total patient cohort experienced complications, as summarized in Figure 1. Notably, hydrothorax emerged as the most prevalent complication, manifesting in 2% of patients undergoing upper pole puncture, while lower pole puncture demonstrated a comparatively lower complication rate.

**Table 2:** Relation between different characteristics and complications in patients

Category	No Complication	Complication	Total N(%)	P-value
	Count (%)	Count (%)		
	N=387(98)	N=13 (2)		
Gender				
Male	211(54.5)	9(75)	220(55)	0.160
Female	176(45.5)	3(25)	179(45)	
Total	387(100)	12(100)	399(100)	
Age (years)				
Median (IQR)	39(29-50)	40(35-51)	39(29-50)	0.442
Min-max	13-90	21-68	11-90	
Side				
Right	197(51)	6(50)	203(51)	0.951
Left	190(49)	6(50)	196(49)	
Total	387(100)	12(100)	399(100)	
Stone burden				
<1.5cm	23(6)	-	23(6)	0.270
1.5 to 2cm	131(34)	3(25)	134(34)	
>2 to 3cm	114(30)	3(25)	117(29)	
>3 to 4cm	49(13)	5(42)	54(14)	
>4cm	8(2)	-	8(2)	
Partial staghorn	30(8)	1(8)	31(8)	
Complete staghorn	32(8)	-	32(8)	
Total	387(100)	12(100)	399(100)	
Degree of obstruction				
Mild	194(50)	6(50)	200(50)	0.335
Moderate	100(26)	4(33)	104(26)	
Severe	10(3)	1(8)	11(3)	
No obstruction	83(21)	1(8)	84(21)	
Total	387(100)	12(100)	399(100)	
Stone location				
Upper Calyce	19(5)	1(8)	20(5)	0.237
Middle Calyce	17(4)	1(8)	18(5)	
Lower calyce	63(16)	-	63(16)	
Pelvis	139(36)	3(25)	142(36)	
Complete staghorn	33(9)	2(17)	35(9)	
Combined	116(30)	5(42)	121(30)	
Total	387(100)	12(100)	399(100)	
Puncture				
Upper	224(58)	9(75)	233(58)	0.446
Lower	159(41)	3(25)	162(41)	
Middle	4(1)	-	4(1)	
Total	387(100)	12(100)	399(100)	
Blood transfusion				
Yes	16(4)	2(17)	18	0.097
No	371(96)	10(83)	381	
Total	387(100)	12(100)	399(100)	
Comorbid conditions				
Hypertension	29(8)	-	29(7)	0.009
Diabetes Mellitus	16(4)	-	16(4)	
Others	45(12)	6(50)	51(13)	
None	297(77)	6(50)	303(76)	
Total	387(100)	12(100)	399(100)	

**Table 3:** Association of demographic and clinical characteristics with stone-free and residual stone after PCNL PCNL: percutaneous nephrolithotomy

Category	Stone-Free N(%) 321(80.5)	Residual Stone N(%) 78(19.5)	Total=399 N(%)	P-value
Age				
Median (IQR)	39(29-50)	38(29-49)		0.936
Min-Max	13-77	13-90		
Gender				
Male	179(56)	41(53)	220	0.614
Female	142(44)	37(47)	179	
Total	321 (100)	78(100)	399 (100)	
Side of the kidney				
Right	170(53)	33(42)	200	0.101
Left	150(47)	45(58)	199	
Total	321 (100)	78(100)	399 (100)	
Stone size				
<1.5cm	20(6)	3(4)	23(5.8)	0.006
1.5 to 2cm	112(35)	22(28)	134(33.6)	
>2 to 3cm	92(29)	25(32)	117(29.3)	
>3 to 4cm	50(16)b	4(5)	54(13.5)	
> 4cm	5(2)	3(4)	8(2)	
Partial staghorn	23(7)	8(10)	31(7.8)	
Complete staghorn	19(6)b	13(17)	32(8)	
Total	321 (100)	78(100)	399(100)	
Degree of obstruction				
Mild	167(52)	33(42)	200	0.365
Moderate	78(24)	26(33)	104	
Severe	9(3)	2(3)	11	
No obstruction	67(21)	17(22)	84	
Total	321 (100)	78(100)	399 (100)	
Location of stone				
Upper Calyx	18(6)	2(3)	20	0.022
Middle Calyx	13(4)	5(7)	18	
Lower Calyx	52(16)	11(14)	63	
Pelvis	114(36)	28(36)	142	
Complete staghorn	21(7)	14(18)	35	
Combined	103(32)	18(23)	121	
Total	321 (100)	78(100)	399 (100)	
Puncture				
Upper	191(59.5)	42(54)	233 (58)	0.507
Lower	127(39.6)	35(45)	162(41)	
Middle	3(0.9)	1(1)	4(1)	
Total	321 (80.5%)	78(19.5%)	399 (100)	

The comparative analysis presented in Table 2 elucidates the demographic and clinical profiles of patients with and without complications. Notably, individuals with comorbidities exhibited a trend toward a higher likelihood of developing complications (P = 0.097). Additionally, a solitary patient encountered a complication associated with a staghorn stone, with half of those experiencing complications also presenting with mild hydronephrosis. Although statistically insignificant, patients experiencing complications tended to necessitate blood transfusions more frequently than their counterparts without complications. Furthermore, lower pole puncture exhibited a diminished complication rate in contrast to upper pole puncture.

Table 3 juxtaposes the demographic and clinical attributes of patients with residual stones against those without. Residual stones were notably more prevalent in cases featuring complete staghorn stones or those exceeding 3-4 centimeters in size, relative to individuals achieving stone-free status post-PCNL. Patients rendered stone-free demonstrated statistically significant localized involvement of the kidney compared to those with residual stones encompassing the entire kidney (P = 0.022). However, age, gender, and the degree of obstruction did not exhibit any significant association with the presence of residual stones. Furthermore, our study revealed a stone clearance rate of 80.5%, including clinically insignificant residual fragments (<5 mm). We defined SFR as the absence of detectable stones on ultrasound and X-ray or the presence of clinically insignificant residual stones (CIRF).

## Discussion

Stone disease presents a significant burden within our nation. Presently, PCNL stands as the foremost treatment modality, rendering open pyelolithotomy largely obsolete in contemporary practice. However, in resource-constrained settings, such as certain developing countries, pyelolithotomy persists due to limitations in equipment availability and a dearth of proficient surgeons trained in advanced endoscopic procedures [13,14]. A variety of percutaneous access techniques have been delineated in the literature, including the "eye of the needle" (bull’s eye) and triangulation methods. Our investigation revealed a preference for the bull’s eye technique for upper pole stones, while the parallel/uniplanar technique or bull’s eye approach was favored for lower pole stones. The selection of a supra 12th puncture site is contingent upon factors such as stone location and renal anatomy, as it affords optimal access to the entire renal calyceal system. Moreover, this approach offers ancillary benefits, facilitating access to the upper ureter and enabling concurrent endopyelotomy [15].

In our study, we observed that complete stone clearance was achieved after the initial attempt in 80.5% of cases. Our findings regarding the success rate of PCNL procedures align with rates reported in existing literature [16-19]. However, our study observed higher residual rates, particularly in cases of partial or complete staghorn stones or when the stone size exceeded 3 cm [20]. Within our study population, 19.7% of patients required additional procedures, with extracorporeal shockwave lithotripsy being the most frequent (n=45, 11.3%), followed by ureterorenoscopy (n=20, 5%), a combination procedure (n=9, 2.3%), and second-look PCNL (n=5, 1.3%). These figures closely align with findings from other studies [21-23]. Biswas et al. reported a stone clearance rate of 82% in a study with 56 patients, demonstrating similarities to our findings [24].

In our study, where both upper and lower pole punctures were performed, we observed a complication rate of 3%. Among the patients, 18 individuals (4.5%) required blood transfusions, while only 2% experienced hydrothorax following an upper pole puncture. Additionally, 0.3% of patients necessitated angioembolization. In contrast, Saeed et al. reported a notably higher complication rate of 33.7%, with 27.5% experiencing bleeding, 4.4% encountering hydrothorax, and 1.9% developing hemothorax. Ullah et al. documented rates of 3.5% for hydrothorax and 7.7% for blood transfusion [15,25]. Comparatively, the incidence of hydrothorax and blood transfusion in these studies exceeded those observed in our investigation. Similarly, Shoaib et al. reported a transfusion rate of 5.63% and one case (0.7%) requiring angioembolization, figures surpassing those observed in our study [19]. In the study by Shahzad et al., complications included failure (4.0%), bleeding (8.57%), fever (55.43%), urinary leakage (8.57%), UTIs (5.14%), colonic injury (0.57%), and nephrectomy necessitated in 0.57% of cases due to severe bleeding. Remarkably, none of the patients in our study experienced urinary leakage or failure, while UTIs were observed in 1% of cases. Although the rate of nephrectomies was similar between our study and Shahzad et al., the incidences of bleeding and fever were notably higher in the latter [26].

The study population had a median age of 39 years with a male preponderance (55%) almost similar to the literature [27]. In our study, 50% of patients displayed mild hydronephrosis, 26% had moderate hydronephrosis, 11% had severe hydronephrosis, and 21% had an undilated system. Among those with mild hydronephrosis, 50% experienced complications. In comparison with the study by Ichaoui et al., our cohort had a higher ratio of mild hydronephrosis and undilated/absent hydronephrosis: 24 (32.4%) with undilated or absent hydronephrosis, 34 (45.9%) with mild hydronephrosis, and 16 (21.6%) with severe hydronephrosis [28].

The median operative time for the 399 PCNL procedures in our study was 60 minutes (IQR: 55-75), irrespective of stone size, including complete staghorn stones. This duration is notably shorter compared to that reported in the existing literature. As surgeon experience increases, there is a potential for reducing operative time. In our procedures, fluoroscopic-guided puncture was utilized.

Our observed median operative time of 60 minutes, regardless of stone size, is noteworthy. Additionally, 50% of patients had a nephrostomy tube in the form of a Nelaton catheter. In contrast, a few authors maintained a nephrostomy tube in the form of a Nelaton catheter in 93.75% of cases, with a mean operative time of 120 min (± standard deviation (SD) 40), indicating superior efficiency in our study. These findings are also favorable compared to those of Thapa et al. [29].

In our study, the majority of patients presented with stones ranging from 1.5 to 4 cm in size. Additionally, 7.7% exhibited partial staghorn stones, while 8% presented with complete staghorn stones. The median age of the study population was 39 years (interquartile range, IQR: 29-50), with a higher proportion being male (55%). Stone distribution revealed a near-equal distribution, with 51% located on the right side and 49% on the left side. In the broader literature, males accounted for 76.87% of cases, with females comprising 23.13%. The average age across studies was reported as 36.4 ± 11.8 years, with a wide range from 18 to 74 years. Moreover, the mean stone size measured 5.7531 ± 18.07 cm³ [9].

Furthermore, nearly half of the patients in our study underwent tubeless PCNL. Notably, as surgical experience increased, an increasing number of patients were able to undergo the procedure without necessitating the use of tubes, a trend consistent with findings in the existing literature [30,31].

The limitations of this study involve it being the experience of a single medical center, which may limit the generalizability of the findings to other settings with different patient populations, surgical expertise, or healthcare systems. The study includes a total of 399 cases. While this number provides a decent sample for analysis, a larger sample size could offer more robust data and reduce the margin of error. This is a retrospective study, there may be inherent biases such as selection bias. Moreover, our study lacks comparative analysis: without a comparison group, such as patients treated with other modalities for renal calculi, it’s difficult to contextualize the efficacy and safety of PCNL relative to alternative treatments.

## Conclusions

In conclusion, our study sheds light on the outcomes of PCNL in a region where such data is scarce. PCNL has emerged as the primary treatment modality for renal stones, surpassing traditional open surgery due to its effectiveness and minimally invasive nature. Our findings demonstrate a high stone clearance rate of 80.5%, which is consistent with existing literature. Notably, PCNL exhibited favorable outcomes even for challenging cases such as staghorn stones and those exceeding 3 cm in size. Complication rates were relatively low, with only 2% experiencing hydrothorax following upper pole puncture and a 3% overall complication rate. Furthermore, our study highlights the importance of surgical expertise in reducing operative time, with a median duration of 60 minutes observed, independent of stone size. Additionally, the transition towards tubeless PCNL reflects advancements in surgical techniques and patient management. Overall, our findings underscore the efficacy and safety of PCNL as the preferred treatment option for renal stones, particularly in resource-constrained settings. Further research and collaborative efforts are warranted to optimize outcomes and expand access to this essential procedure.
